# Supplementary material for: Reduction in global area burned and wildfire emissions since 1930s enhances carbon uptake by land
Source: Nat Commun. 2018 Apr 17;9:1326. doi: 10.1038/s41467-018-03838-0 (PMC5904128; doi:10.1038/s41467-018-03838-0)
Supplement: Supplementary file 1 — Supplementary Information [file 41467_2018_3838_MOESM1_ESM.pdf]

Supplementary information for “Reduction in global area burned and wildfire emissions since 1930s enhances carbon uptake by land” authored by Vivek Arora and Joe Melton

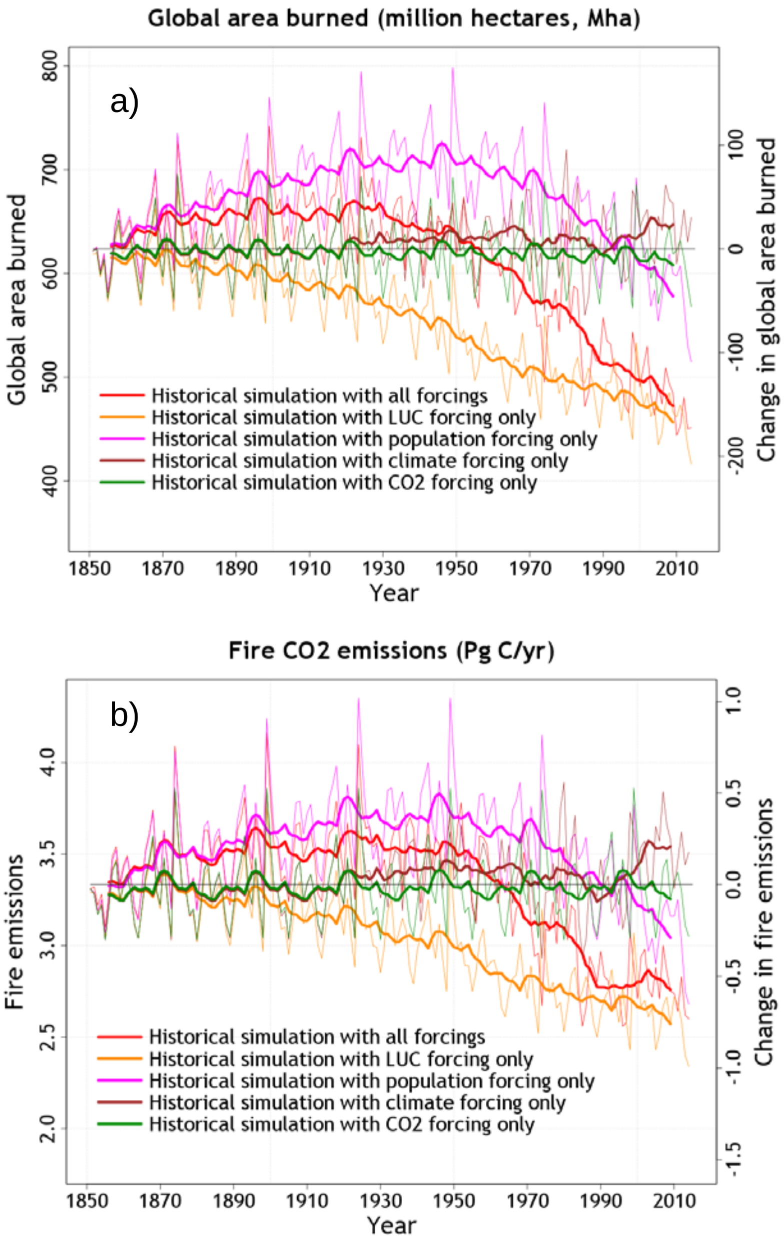

Supplementary Figure 1: Simulated global burned area and fire CO_2 emissions. Similar to Figure 1 but area burned (panel a) and fire CO_2 emissions (panel b) are also shown from simulations driven with climate and CO_2 forcings individually. Changes in population and land cover are the biggest drivers of change in area burned and fire CO_2 emissions over the historical period. The thick lines are the 10-year moving averages.

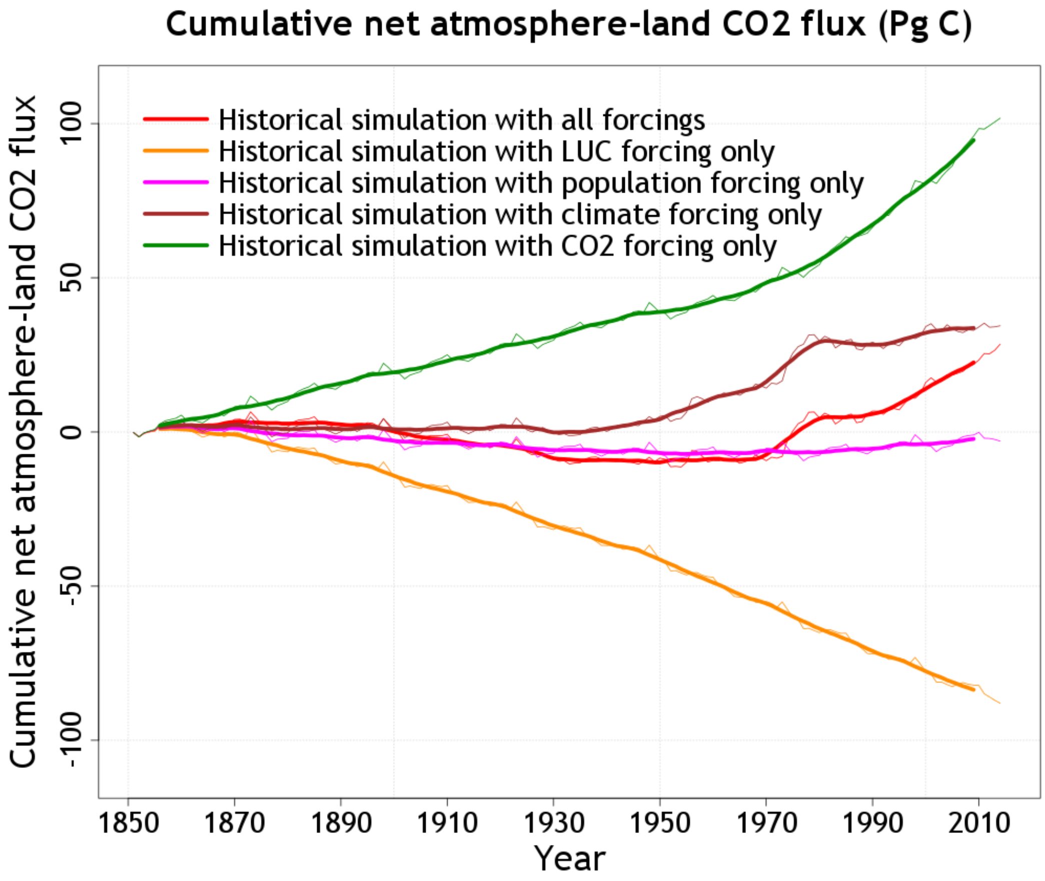

Supplementary Figure 2: Simulated cumulative net atmosphere-land CO_2 flux. Results are shown from five simulations. The first simulation includes the effect of all forcings while the remaining four include the effect of individual forcings and are driven by changes in CO_2, land use change, population density, and climate over the historical period. The thick lines are the 10-year moving averages.

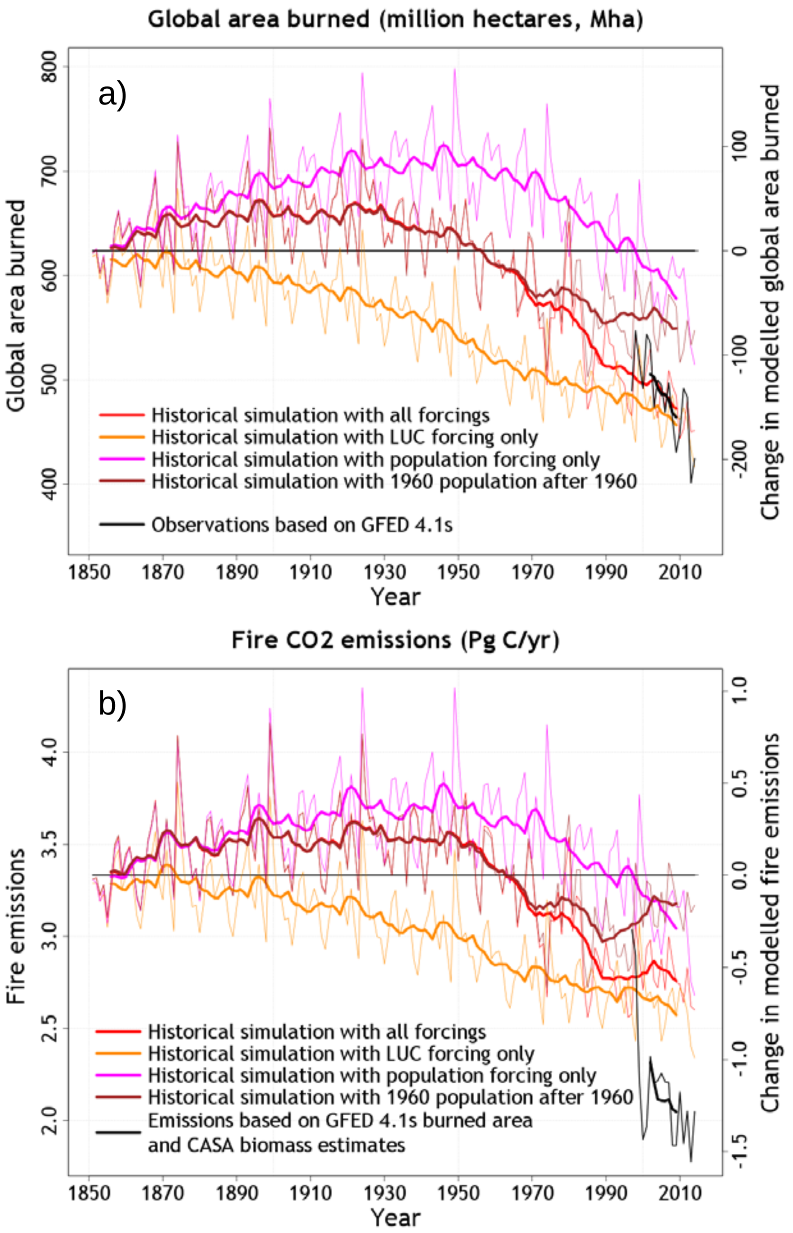

Supplementary Figure 3: Similar to Figure 1 but area burned (panel a) and fire CO_2 emissions (panel b) are also shown from the simulation in which population densities stay at their 1960 levels after 1960 (brown line). As expected then, the area burned and fire CO_2 emissions do not reduce as much as in the historical simulation with all forcings (red line). The cumulative difference in fire CO_2 emissions between these two simulations (the red and brown lines) then yields the impact of population changes since 1960 on the land carbon budget and is calculated to be 7.8 Pg C for the 1960-2009 period. That is, population changes since 1960 decrease global fire emissions.

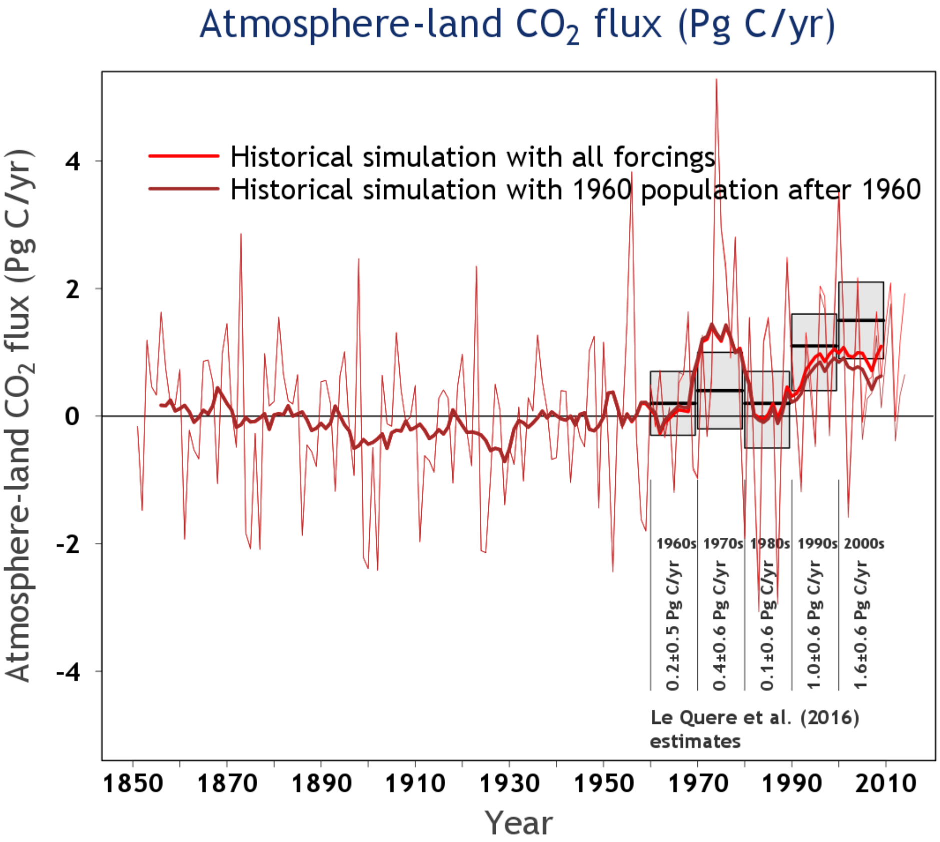

Supplementary Figure 4: Simulated net atmosphere-land CO_2 flux from the 1851-2014 historical simulation that includes all forcings and the simulation in which population densities are kept at their 1960 levels after 1960. The thick line is the 10-year moving average value. The net atmosphere-land CO_2 flux is higher in the historical simulation with all forcings because the continually increasing population densities yield a decrease in fire emissions which increases the carbon sink over land. Simulated results are also compared with estimates from the Global Carbon Project (Le Quere et al., 2016, ref. 3). Simulated fluxes lie within the uncertainty range of observation-based estimates except during 1970s.

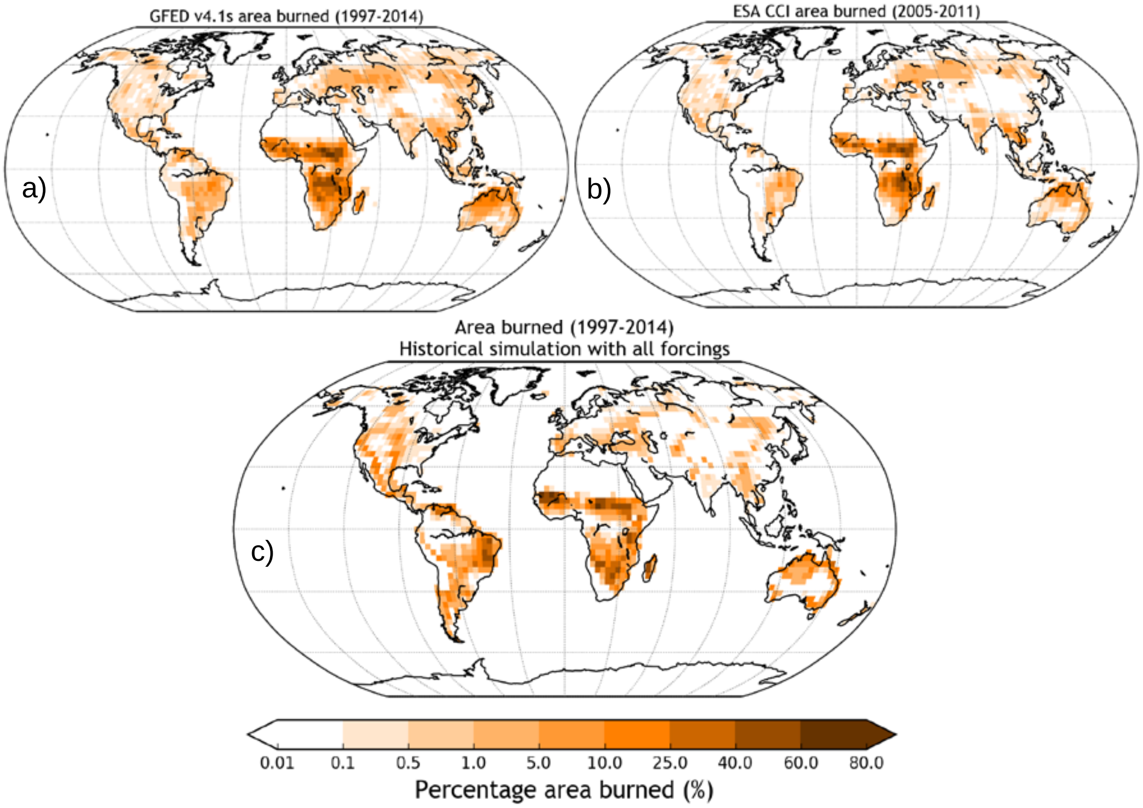

Supplementary Figure 5: Comparison of geographical distribution of simulated area burned for the period 1997-2014 (panel c) with the two satellite-based estimates – GFED4.1s[14,15] for the period 1997-2014 (panel a) and ESA CCI[27] for the period 2005-2011 (panel b).

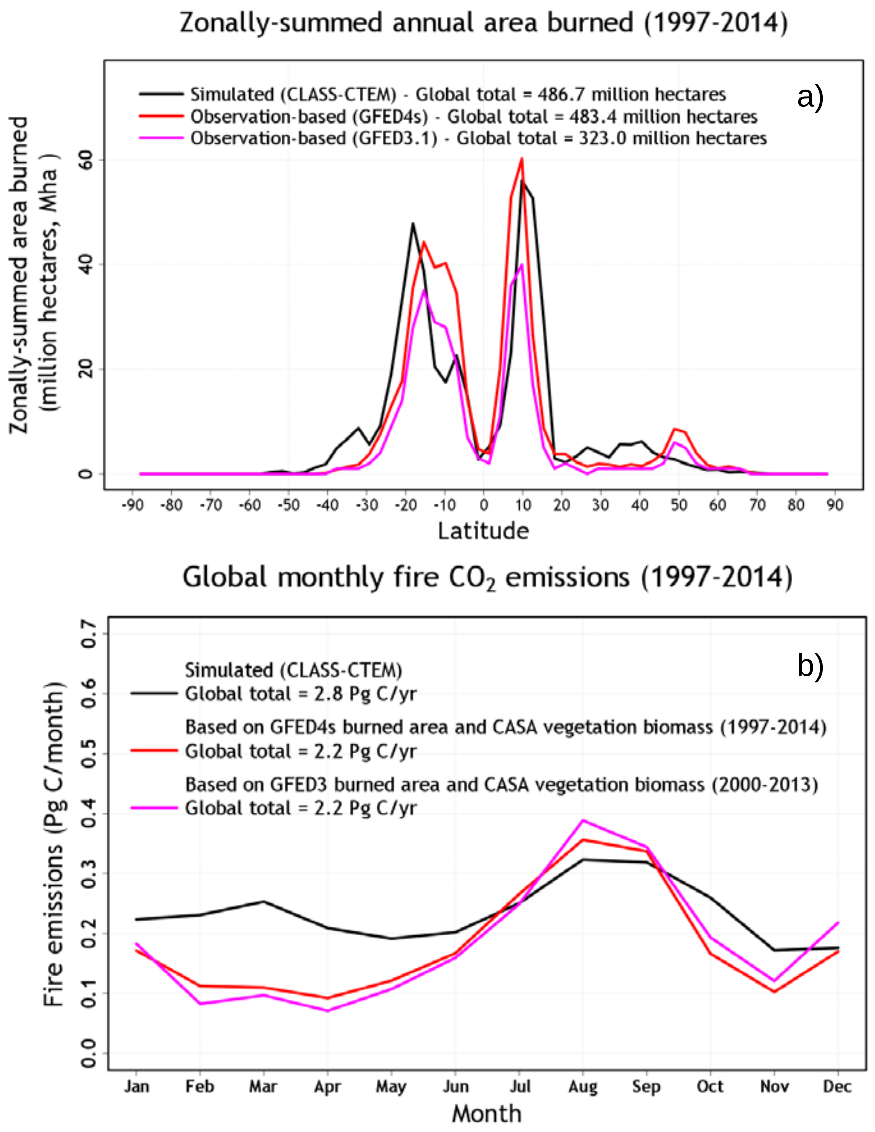

Supplementary Figure 6: Comparison of the latitudinal distribution of simulated area burned with data from version 4.1s (refs [14,15]) and 3 (ref. [31]) of the GFED (panel a). Panel (b) compares the seasonality of simulated global fire emissions with estimates from the GFED versions 4.1s and 3.

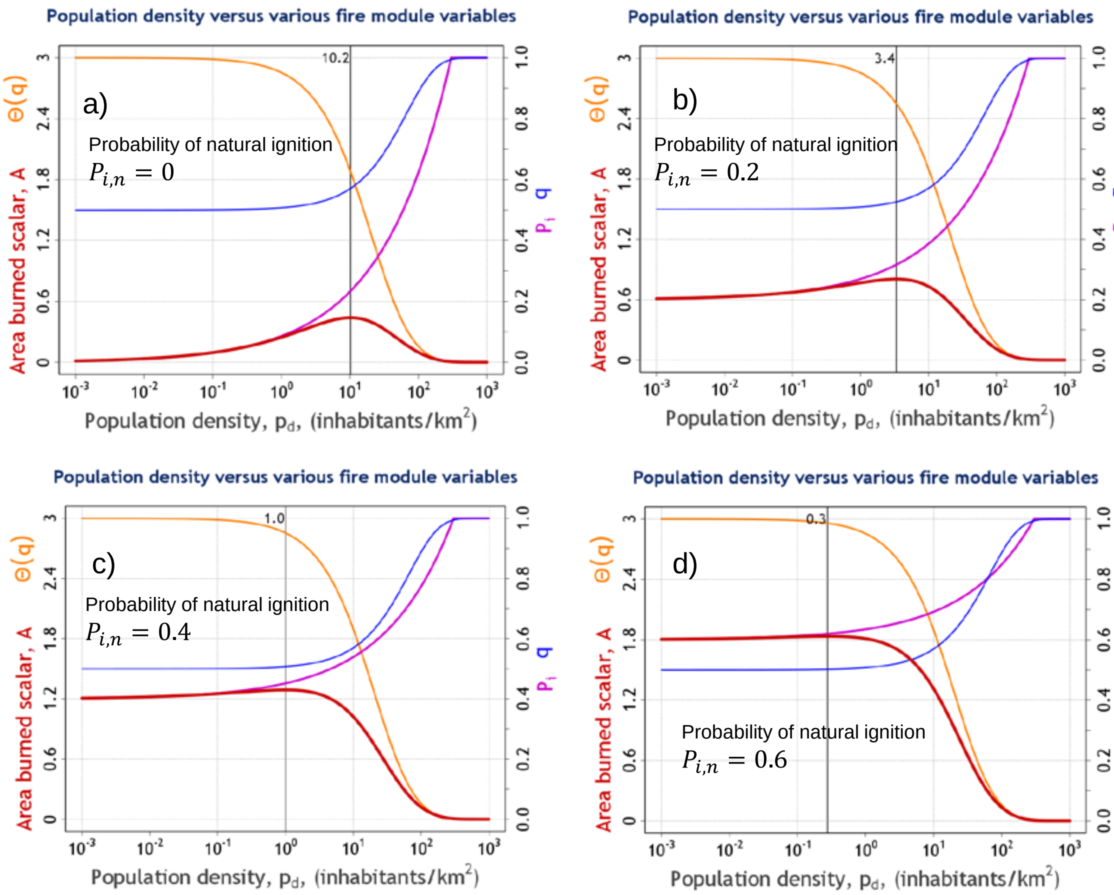

Probability of human-caused fire ignitions $P_{i,h} = \max\left(1, \left(\frac{p_d}{300}\right)^{0.43}\right)$ (1)

Probability of ignition $P_i = P_{i,n} + (1 - P_{i,n})\, P_{i,h}$ (2)

Fire extinguishing probability $q = \left(0.5 + 0.5\left(1 - \exp(-0.025 p_d)\right)\right)$ (3)

Fire duration $\tau = (1 - q)/q$ (4)

Area burned multiplier $\Theta(q) = (1 - q)(2 - q)/q^2$ (5)

Area burned scalar, $A(q, P_{i,h}, P_{i,n})$ $A = \Theta(q) P_i$ (6)

Supplementary Figure 7: Dependence of area burned on population density and probability of fire conditioned on natural ignition (P_n). The fire module of the CLASS-CTEM model is explained in detailed in Melton and Arora (2016)[17]. The figure shows the basis of the model behaviour which results in area burned (dark red line) first increasing as population density increases but, as direct suppression of fire and landscape fragmentation increase (both of which are modelled as a function of population density), area burned begins to decrease. The net result is that there is an optimum population density (10.2 inhabitants/km^2) at which the area burned is maximized for P_n=0. This is shown in panel (a). However, as ignition due to lightning increases then the increase in population density primarily reduces area burned (panels b, c and d).

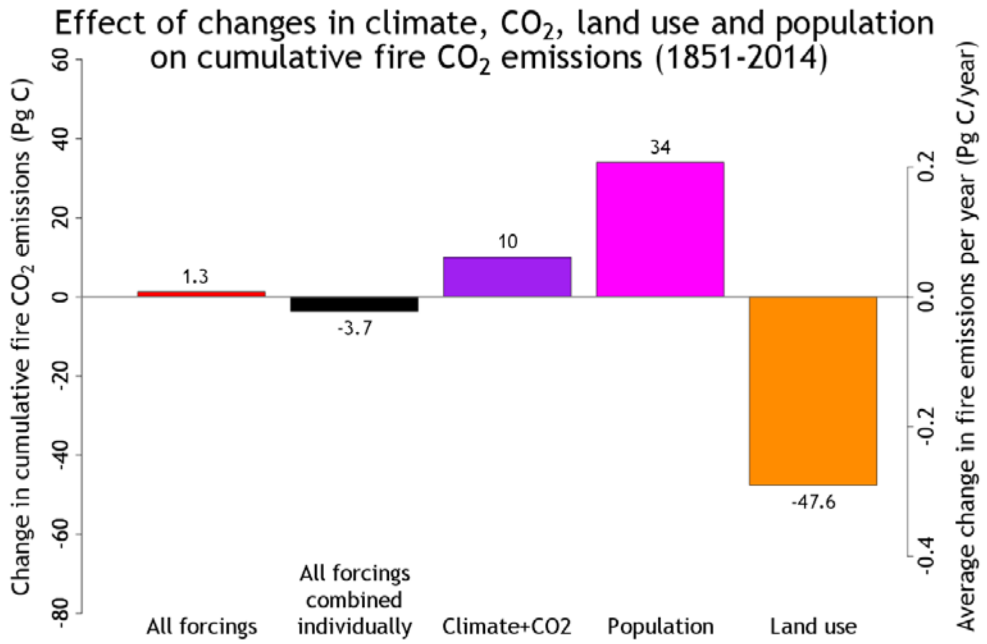

Supplementary Figure 8: The effect of individual forcings on fire CO_2 emissions for the period 1851-2014 based on Supplementary Figure 1 panel (b). The secondary y-axis is equal to the cumulative amount divided by 164 (equal to number of years in the 1851-2014 period). Over the 1851-2014 period changes in population density contribute to an overall increase in fire emissions, changes in land cover lead to decrease in fire emissions, and combined climate change and the increase in CO_2 lead to a small increase in fire emissions. The individual contributions, calculated from simulations with individual forcings, do not sum together to yield the same decrease in cumulative fire CO_2 emissions as the simulation with all forcings because of the spatial correlations between different forcings (e.g. crop area increase correlates positively with population increases) but also the non-linear response of the model to individual forcings.
